# Mitochondria of a human multidrug-resistant hepatocellular carcinoma cell line constitutively express inducible nitric oxide synthase in the inner membrane

**DOI:** 10.1111/jcmm.12528

**Published:** 2015-02-18

**Authors:** Ornella Fantappiè, Chiara Sassoli, Alessia Tani, Daniele Nosi, Serena Marchetti, Lucia Formigli, Roberto Mazzanti

**Affiliations:** a Department of Experimental and Clinical Medicine – Section of Internal Medicine and Hepatology, University of Florence and Azienda Ospedaliero Universitaria CareggiFlorence, Italy; b Department of Experimental and Clinical Medicine – Section of Anatomy and Histology, University of FlorenceFlorence, Italy; c Department of Experimental Therapy and Medical Oncology, The Netherlands Cancer InstituteAmsterdam, The Netherlands

**Keywords:** MDR, iNOS, mitochondrion, COX-2, coxib

## Abstract

Mitochondria play a crucial role in pathways of stress conditions. They can be transported from one cell to another, bringing their features to the cell where they are transported. It has been shown in cancer cells overexpressing multidrug resistance (MDR) that mitochondria express proteins involved in drug resistance such as P-glycoprotein (P-gp), breast cancer resistant protein and multiple resistance protein-1. The MDR phenotype is associated with the constitutive expression of COX-2 and iNOS, whereas celecoxib, a specific inhibitor of COX-2 activity, reverses drug resistance of MDR cells by releasing cytochrome c from mitochondria. It is possible that COX-2 and iNOS are also expressed in mitochondria of cancer cells overexpressing the MDR phenotype. This study involved experiments using the human HCC PLC/PRF/5 cell line with and without MDR phenotype and melanoma A375 cells that do not express the MDR1 phenotype but they do iNOS. Western blot analysis, confocal immunofluorescence and immune electron microscopy showed that iNOS is localized in mitochondria of MDR1-positive cells, whereas COX-2 is not. Low and moderate concentrations of celecoxib modulate the expression of iNOS and P-gp in mitochondria of MDR cancer cells independently from inhibition of COX-2 activity. However, A375 cells that express iNOS also in mitochondria, were not MDR1 positive. In conclusion, iNOS can be localized in mitochondria of HCC cells overexpressing MDR1 phenotype, however this phenomenon appears independent from the MDR1 phenotype occurrence. The presence of iNOS in mitochondria of human HCC cells phenotype probably concurs to a more aggressive behaviour of cancer cells.

## Introduction

P-glycoprotein (P-gp), multidrug-resistant protein 1 (MRP1) and breast cancer resistant protein (BCRP) are membrane proteins belonging to the family of multidrug-resistant (MDR) proteins that mediate intrinsic and acquired drug resistance in cancer cells. Malignant tumours that possess the MDR phenotype are more resistant to several unrelated anticancer drugs, such as doxorubicin, vinca alkaloids, taxanes and others, and cause chemotherapy failure. The MDR phenotype is a complex phenomenon and these proteins, also called ABC transporters, mainly expressed in the plasma membrane and acting by reducing the intracellular drug concentration, are mediators of the phenotype. Nevertheless, the functions of P-gp, MRP1 and BCRP are probably multiple, with some of them still unknown. It must also be remembered that these proteins are also expressed in several normal tissues such as liver, kidney, gastrointestinal tract, placenta and blood–brain barrier, where they play an important role in the physiology of these tissues, by protecting them from toxic xenobiotics and endogenous metabolites 1–3.

In recent years, the localization of these proteins on the nuclear envelope and on the membrane of cytoplasmic organelles, such as mitochondria and endoplasmic reticulum, has suggested their involvement in several signalling pathways 4–10. It has been shown that P-gp activity is related to the expression of the inducible isoform of the cyclooxygenase-2 (COX-2) enzyme. The cyclooxygenase pathway plays an important role in the regulation of inflammation and cancer, and COX-2 appears to be important in tumour growth, metastasis process and tumour drug resistance 11,12. In addition, a relationship has been found between COX-2 and BCRP activity and between COX-2 and MRP1 13,14. We have demonstrated that MDR1-positive cancer cells express constitutively COX-2 and that celecoxib, a specific inhibitor of COX-2 activity, can reverse drug resistance to apoptosis of these cells by a P-gp-dependent but COX-2-independent mechanism in MDR1-positive cells 15,16.

The occurrence of the MDR1 phenotype in cancer cells includes the expression of inducible nitric oxide synthase (iNOS) and production of nitric oxide that is greater than that in a parental drug-sensitive cell line 15. Higher expression of iNOS leads to increased angiogenic activity of an MDR1-positive HCC cell line, confirming the observation that nitric oxide and proteins involved in drug resistance such as P-gp, BCRP or MRP1, are linked to each other at least in this cancer cell line 17–19.

Nitric oxide is a small highly reactive lipophilic molecule that regulates several pathophysiological processes inside the cells. It can easily diffuse in water and also through cell membranes and its high reactivity limits its half-life in biological fluids, influencing its localization, concentration and duration of activity 20. The complexity of its biological effects explains the controversial results obtained by different research groups. Nitric oxide regulates cell respiration, inhibits cytochrome C oxidase, and reduces O_2_ consumption 21. Nitric oxide produced in normal conditions by the constitutive NOS isoforms, nNOS and eNOS, does not reach concentrations that can significantly inhibit respiration. However, iNOS can produce high amounts of nitric oxide and for prolonged periods of time, damaging a variety of cellular targets and leading to apoptosis and mutagenesis 21.

The role of nitric oxide in apoptosis is controversial as it can be pro-apoptotic or anti-apoptotic 22. It has been shown that nitric oxide-dependent apoptosis (that probably has antitumour activity) requires high concentrations of nitric oxide while low concentrations of it can protect cells from undergoing apoptosis, likely favouring tumour growth 23. It must be said that nitric oxide can regulate two vital cell processes: energy production and apoptosis, and in both pathways mitochondria play a crucial role 24,25.

For these reasons, we decided to study whether P-gp and BCRP, both involved in conferring drug resistance, are expressed in mitochondrial proteome. The mitochondrial proteome includes several proteins of unknown function 26. We have demonstrated that P-gp and BCRP are overexpressed and functionally active in the mitochondrial cristae of an MDR1-positive cancer cell line, and Roundhill *et al*. have shown the presence of MRP1 with efflux activity in human mitochondria 4,6,9. The observation that P-gp, BCRP and MRP1 are localized also in mitochondria of MDR1-positive cancer cells could explain why drug-resistant cells do not release cytochrome c into cytosol and the P-gp mediated celecoxib-induced apoptosis 16,27. Taken together, these findings suggest that the presence of the most important drug-resistant proteins in mitochondria of MDR1 cancer cells is not casual but causative. As mitochondria are involved in cell apoptosis, it is possible to postulate that COX-2 and iNOS are expressed in these organelles. This study addresses the question of whether COX-2 and iNOS can be localized in mitochondria of MDR1 cancer cells and whether drugs that are known to affect apoptosis can modulate their expression.

## Materials and methods

### Cell lines

Experiments were performed on a human HCC cell line PLC/PRF/5 28 by using parental drug-sensitive clone (P5) and the highly MDR1 subclone (P1(0.5)). The P1(0.5) subclone was developed by prolonged serial exposures to increasing concentrations of doxorubicin (DOX) (Santa Cruz Biotechnology, CA, USA) starting from parental P5 cells, and subsequently cultured in DMEM containing 0.5 μg/ml DOX and 10% foetal bovine serum (FBS), as previously reported 15. Confirmative experiments were done in melanoma cell line A375 (American Type Culture Collection, ATCC, Manassas, VA, USA) that express iNOS but do not express the MDR1 phenotype. A375 cells were cultured in DMEM and 10% of FBS. All cell lines were cultured at 37°C in an atmosphere containing 5% CO_2_.

### Cell treatment

Because only some celecoxib (added to medium containing 10% FBS) is not protein-bound and hence available for interacting on cells, we used DOX-free, serum-free medium containing celecoxib in all experiments, as described in the details for each experimental condition 29. Cells were seeded in complete medium for 24 hrs and then exposed to DOX-free, serum-free medium containing 2.5, 5, 10, 20 or 50 μmol/l celecoxib (Sigma-Aldrich, Milan, Italy). After 6 hrs, total proteins were extracted and evaluated by Western Blot analysis. In other experiments, MDR1-positive P1(0.5) HCC cells were exposed to DOX-free, serum-free medium containing 10–50 μmol/l celecoxib for 6 hrs and then were processed for confocal immunofluorescence microscopy analysis and Western blot analysis.

### Western blot analysis

Preparation of total protein lysates and Western blot analysis was performed as previously described 15, using each of the following primary antibodies: anti-COX-2 polyclonal antibody (Santa Cruz Biotechnology), anti-P-gp (C219) monoclonal antibody (Gene Tex, Irvine, CA, USA), anti-iNOS polyclonal antibody (Santa Cruz Biotechnology), anti-BCRP monoclonal antibody (Chemicon, Temecula, CA, USA), anti-apoptosis-inducing factor (AIF-H300) polyclonal antibody (Santa Cruz Biotechnology), anti-heat shock protein 60 (HSP-60) polyclonal antibody (Santa Cruz Biotechnology) and anti-β-actin monoclonal antibody (Sigma-Aldrich).

### Preparation of mitochondrial fractions

Cells were washed (PBS, 3×), resuspended in homogenization buffer (HB; 150 mmol/l MgCl_2_, 10 mmol/l KCl, 10 mmol/l Tris, pH 6.7, protease inhibitors; 1 ml/1 × 10^7^ cells), incubated on ice (15 min.), and Dounce homogenized (35 strokes) (WCS: whole cell sample). HB with sucrose (34.2%, 1/3 vol.) was added and centrifuged (‘low’ speed, 1000 × g, 5 min., 4°C) to remove nuclei and unlysed cells. The pellet, containing nuclei and unlysed cells (LSP: low speed pellet) was resuspended in mitochondrial suspension buffer (250 mmol/l sucrose, 10 mmol/l Tris, pH 7.0 with protease inhibitors). The supernatant was centrifuged (‘medium’ speed, 5000 × g, 10 min., 4°C), the pellet (CM: crude mitochondria) was resuspended in HB + sucrose (20 ml), divided into two samples and centrifuged (5000 × g, 10 min., 4°C). One resultant pellet was resuspended in Solution A (3 ml; 20 mmol/l HEPES, 1 mmol/l EDTA, 250 mmol/l sucrose, pH 7.4). Iodixanol solution (50% iodixanol, 120 mmol/l HEPES, 6 mmol/l EDTA, 250 mmol/l sucrose, pH 7.4) was added (final concentration of 36%), placed in a centrifuge tube, overlayed with Solution B (10 ml, 30% iodixanol, 80 mmol/l HEPES, 4 mmol/l EDTA, 250 mmol/l sucrose, pH 7.4), then Solution C (to top, 10% iodixanol, 80 mmol/l HEPES, 4 mmol/l EDTA, 250 mmol/l sucrose, pH 7.4), and centrifuged (50,000 × g, 4 hrs, 4°C, swinging bucket rotor). Protein was collected at the 30%/10% iodixanol interface, an equal volume of Solution A (10 ml) was added, followed by centrifugation (30,000 × g, 10 min., 4°C). The resulting pellet was resuspended in mitochondrial suspension buffer (250 mmol/l sucrose, 10 mmol/l Tris, pH 7.0) with protease inhibitors (MFI: mitochondrial fraction iodixanol). The second CM pellet was resuspended in resuspension buffer (RB = 10 mmol/l TRIS, 0.5 mmol/l EDTA, 10% glycerol, pH 7.5), applied to the top of a sucrose gradient (53.5% sucrose/43.5% sucrose), centrifuged (5 hrs, 100,000 × g, 4°C) and the resulting fraction at the 53.5/43.5% sucrose interface was collected, diluted (RB, 1 vol.) and centrifuged (120,000 × g, 1 hr, 4°C). The pellet was resuspended in TSNa buffer (10 mmol/l TRIS, pH 7.5, 50 mmol/l NaCl, 250 mmol/l sucrose) with protease inhibitors (MFS: mitochondrial fraction sucrose) 30.

### Confocal immunofluorescence

Living parental drug-sensitive P5, celecoxib untreated and treated MDR1-positive P1(0.5) HCC cells and A375 melanoma cells grown on glass coverslips were incubated for 30 min. at 37°C with 100 nmol/l of MitoTracker red CMXRos (Molecular Probes Inc., Eugene, OR, USA), a cell-permeable mitochondria selective dye, fixed in 0.5% buffered paraformaldehyde for 10 min. at room temperature (RT) and then processed for confocal immunofluorescence examination of iNOS. After permeabilization with cold acetone for 3 min., washed cells were incubated in blocking solution [0.5% bovine serum albumin (Sigma-Aldrich) and 3% glycerol in PBS] for 30 min. and then with rabbit polyclonal anti-iNOS (1:200; Santa Cruz Biotechnology) for 1 hr at RT. The immunoreactions were revealed by incubation of the cells with goat anti-rabbit Alexa Fluor 488-conjugated IgG (Molecular Probes Inc.). Replacing the primary antibodies with non-immune rabbit serum negative controls were carried out; cross-reactivity of the secondary antibodies was tested in control experiments in which primary antibodies were omitted. Finally, the coverslips containing the immunolabelled cells were mounted with an antifade mounting medium (Biomeda Gel mount, Electron Microscopy Sciences, Foster City, CA, USA) and then viewed under a confocal Leica TCS SP5 microscope (Leica Microsystems, Mannheim, Germany) equipped with an HeNe/Ar laser source for fluorescence measurements. The observations were performed with a Leica Plan Apo 63X/1.43NA oil immersion objective. Series of optical sections (1024 × 1024 pixels each; pixel size 204.3 nm), 0.4 μm in thickness, were taken through the depth of the cells at intervals of 0.4 μm. Images were then projected onto a single ‘extended focus’ image. Qualitative assessment of colocalization between iNOS and MitoTracker fluorescence signals was performed by Image J colocalization Plugin Software (NIH, http://imagej.nih.gov/ij/) and the quantitative analysis was done by calculating the overlap coefficient (ranging from 0, minimum colocalization degree, to 1, maximum colocalization degree) using JACoP Plugin Image J 31. At least 50 different cells were analysed in each experiment (*n* = 3). Calculations were performed with GraPhPad Prism software program (GraphPad, San Diego, CA, USA).

### Immunogold electron microscopy

Cells were detached and centrifuged at 895 g for 10 min.; the pellets were fixed in 4% PFA for 1 hr, dehydrated and embedded in Epon 812 essentially as reported previously 6. Ultra-thin sections collected on nickel grids were etched with 30% hydrogen peroxide for 10 min., incubated with normal goat serum (1:25 in TBS, pH 7.6; Sigma) for 15 min. to quence a non-specific binding site and then with rabbit polyclonal anti-iNOS (1:200; Santa Cruz Biotechnology) overnight at 4°C. After washing in TBS (pH 7.6 and 8.2), the immunoreaction was revealed by incubating the sections with anti-rabbit IgG antibodies conjugated with 10 nm colloidal gold particles [1:25 in TBS (pH 8.2), BB International, Cardiff, UK] overnight at 4°C. Negative controls were carried out by replacing the primary antibody with non-immune serum. Finally, the ultra-thin sections were counterstained with uranyl acetate for 10 min. and observed under a Joel 1010 (Jeol, Tokyo, Japan) transmission electron microscope at 80 kV.

## Results

### Expression of COX-2 and iNOS in mitochondrial fractions in MDR1-positive P1(0.5) cells

**Figure 1:**
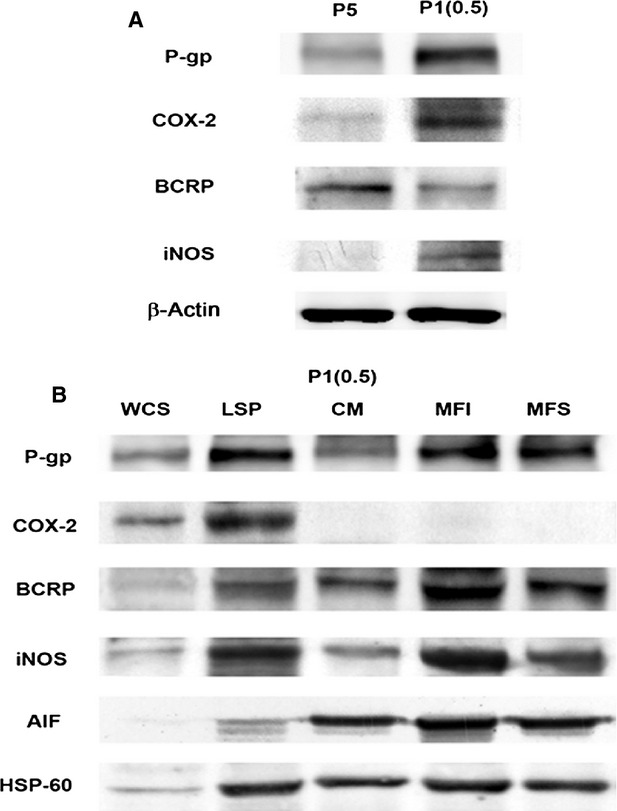
Expression of P-gp, COX-2, BCRP and iNOS in P5 and MDR1-positive P1(0.5) cells. (A) Western blot analysis of P-gp, COX-2, BCRP and iNOS expression in total cell lysate of P5 and P1(0.5) cells at basal conditions. β-actin was used as a protein-loading control. (B) P-gp, COX-2, BCRP and iNOS protein expression in different fractions of P1(0.5) cells obtained during the preparation of mitochondria by differential centrifugation following purification on an iodixanol or sucrose gradient as described in the Materials and methods section. WCS: whole cell lysate; LSP: low speed pellets; CM: crude mitochondria; MFI: mitochondrial fraction iodixanol; MFS: mitochondrial fraction sucrose. AIF and HSP-60 were used as mitochondrial marker proteins. One representative experiment out of three performed is shown.

**Figure 2:**
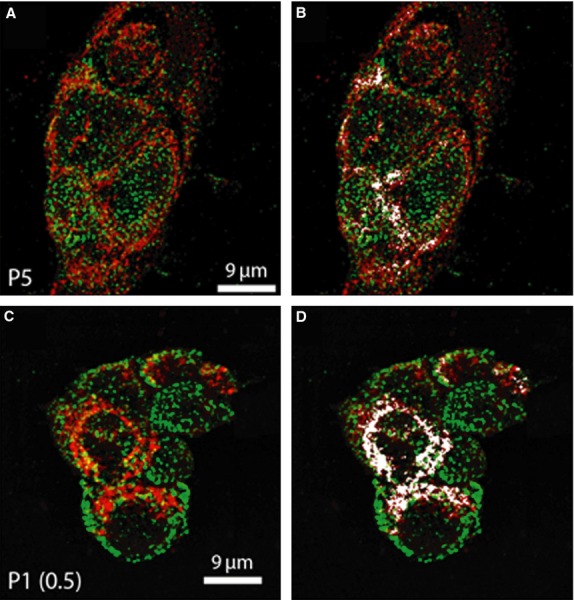
Confocal immunofluorescence analysis of mitochondrial localization of iNOS in P5 and MDR1-positive P1(0.5) cells. Representative confocal immunofluorescence images of (A) P5 and (C) MDR1-positive P1(0.5) cells grown on glass coverslips, incubated with 100 nmol/l of MitoTracker red CMXRos to label mitochondria (red), fixed and immunostained for iNOS expression (green). Note the colocalization between the two fluorescent signals (yellow) indicating the mitochondrial localization of the iNOS. (B and D) Qualitative assessment of the colocalized points (white) obtained by using Image J colocalization Plugin Software (NIH). The images are representative of at least three separate experiments with similar results.

Before studying the expression of COX-2 and iNOS in mitochondria, we searched for the expression of P-gp, COX-2, BCRP and iNOS in MDR1-positive P1(0.5) cells as compared to parental drug-sensitive P5 cells (Fig.1A). Western blot analysis shows the presence of P-gp and BCRP in mitochondrial fractions of P1(0.5) cells (Fig.1B). In addition, it reveals that MDR1-positive P1(0.5) cells constitutively express P-gp, COX-2 and iNOS proteins. These proteins were not expressed in parental drug-sensitive P5 cells (Fig.1A). BCRP, as expected, was expressed both in parental-sensitive and MDR1 cell lines, and it was slightly more expressed in P5 cells (Fig.1A). To investigate the presence of COX-2 and iNOS in mitochondria, pure fractions of mitochondria were prepared by differential density gradient centrifugation using density media – iodixanol or sucrose (see Materials and methods), and the fractions of each centrifugation step were analysed by Western blot (Fig.2A). AIF, a protein found primarily in the mitochondria, was used as a marker for the purity of mitochondrial preparations 32. Data shown in this work confirm the mitochondrial localization of P-gp and BCRP in MDR1 cancer cells and, most importantly, show the presence of iNOS in mitochondrial fractions, whereas COX-2 was not expressed (Fig.1B).

### Mitochondrial localization of iNOS in MDR1-positive P1(0.5) cells by confocal and electron microscopy analyses

**Figure 3:**
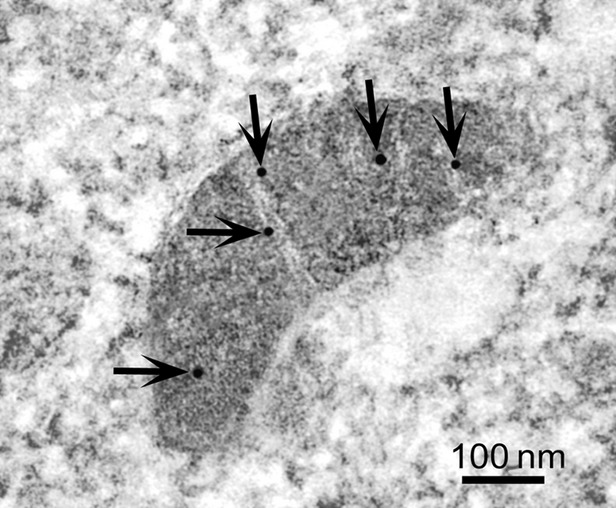
Immunoelectron cytochemistry analysis of iNOS protein in MDR1-positive P1(0.5) cells. Representative transmission electron microscopy image of Epon-812-embedded samples of MDR1-positive P1(0.5) cells processed for immunogold labelling of anti-iNOS antibody. The photograph shows a mitochondrion with the cristae decorated with iNOS-gold particles (arrows). The image represents at least three separate experiments with similar results.

Confocal and immunoelectron microscopic analyses of the iNOS were carried out to detect the precise subcellular localization of the protein in both parental drug-sensitive P5 and their derived MDR1-positive P1(0.5) intact cells. Using confocal microscopy (Fig.2), we found that iNOS was robustly expressed in MDR1 P1(0.5) and parental drug-sensitive P5 cell clones at level of plasma membrane and in the cytoplasm with a clear mitochondrial localization. The results of the colocalization analysis between the fluorescent signals relative to iNOS and mitochondria, performed by using JACoP Plugin Image J (NIH), indicated an overlap coefficient between the two signals of 0.475 ± 0.05 in P5 and 0.524 ± 0.01 in P1(0.5) cells, confirming the expression of iNOS at the mitochondrial level. Immunoelectron microscopy studies revealed that iNOS was localized at the level of the mitochondrial cristae (Fig.3).

### Celecoxib modulates the expression of iNOS

**Figure 4:**
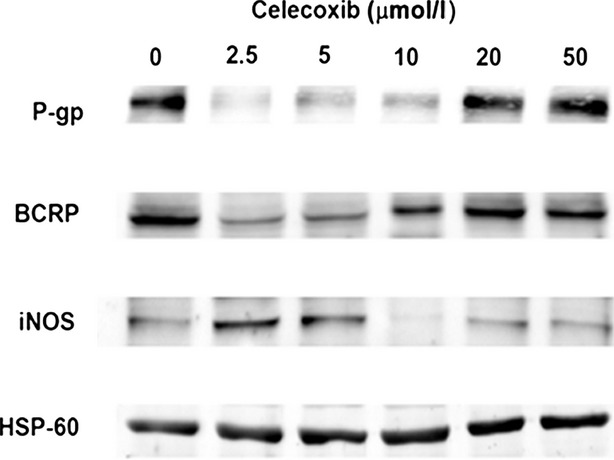
Effect of celecoxib on the expression of P-gp, BCRP and iNOS in mitochondria of MDR1-positive P1(0.5) cells. Mitochondria of P1(0.5) cells were purified by using the fraction methodology based on iodixanol separation as described in the Materials and methods section. HSP-60 were used as a protein-loading control. One experiment representative of the three performed is shown.

**Figure 5:**
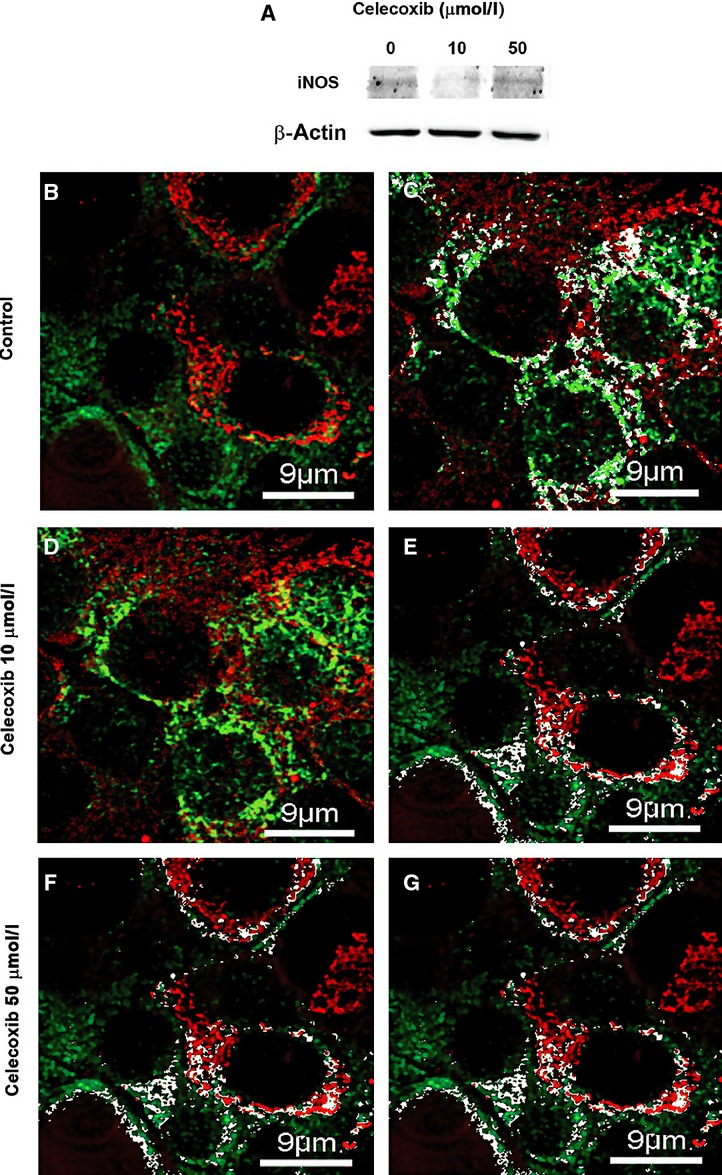
Analysis of iNOS expression in MDR1-positive P1(0.5) cells cultured at basal condition (0) and after treatment with 10 or 50 μmol/l celecoxib for 6 hrs. (A) Western blot analysis of iNOS in total cell lysate. β-actin was used as a protein-loading control. (B–G) Representative confocal immunofluorescence images of MDR1-positive P1(0.5) cells grown on glass coverslips incubated with 100 nmol/l of MitoTracker red CMXRos to label mitochondria (red) fixed and immunostained for iNOS expression (green). The colocalization between the two fluorescent signals (yellow) indicates the mitochondrial localization of the iNOS. In C, E, G, qualitative assessment of the colocalized points (white) between the two fluorescent signals obtained by using Image J colocalization Plugin Software (NIH) is shown. Note that the treatment with 10 μM/l celecoxib (D and E) reduced iNOS expression as compared with the untreated cells (B and C), whereas the treatment with 50 μM/l celecoxib (F and G) did not modify its expression. The localization of iNOS at mitochondrial level did not vary upon celecoxib treatment (C, E and G). The images are representative of at least three separate experiments with similar results.

To evaluate a possible association between P-gp expression and BCRP or iNOS level in mitochondria of MDR1-positive P1(0.5) cells, the expression of these proteins was determined in mitochondrial fractions of P1(0.5) cells cultured for 6 hrs in a doxorubicin-free, serum-free medium containing 2.5, 5, 10, 20 or 50 μmol/l celecoxib. We have previously shown that 10 μmol/l celecoxib markedly reduced the expression of P-gp, whereas 50 μmol/l did not affect P-gp expression in whole cell lysate of P1(0.5) cells 16. Western blot analysis clearly showed that the expression of P-gp and BCRP was reduced in mitochondrial fractions of P1(0.5) cells treated for 6 hrs with 2.5 or 5 μmol/l celecoxib. On the contrary, the expression of iNOS was increased under the same experimental conditions (Fig.4). Cell exposure to 10 μmol/l celecoxib reduced mitochondrial P-gp and iNOS expression and did not alter the BCRP mitochondrial level (Fig.4). The higher concentrations of celecoxib, 20 or 50 μmol/l, did not modify the mitochondrial expression of P-gp, BCRP or iNOS in P1(0.5) cells (Fig.4). To complete the study of celecoxib effect on modulation of iNOS expression in P1(0.5) cells, we tested the expression of iNOS in total cell lysate of P1(0.5) cells cultured at basal condition and after treatment with 10 or 50 μmol/l celecoxib for 6 hrs. Western blot analysis showed that 10 μmol/l celecoxib reduced the iNOS expression in total cell lysate, whereas 50 μmol/l did not (Fig.5A).

Confocal immunofluorescence analysis of iNOS expression in MDR1-positive P1(0.5) cells confirmed the results obtained by Western Blotting showing a decrease in the expression of the protein after treatment with celecoxib 10 μM/l as compared to untreated cell, whereas celecoxib 50 μM/l did not modify its expression (Fig.5B, D and F). The overlap coefficient between the fluorescent signals relative to iNOS and mitochondria did not vary upon celecoxib treatment (0.52 ± 0.07 in untreated and 0.51 ± 0.04 in treated cells). These data suggested that the decrease in iNOS expression induced by celecoxib correlates with a global decrease in iNOS expression rather than with its intracellular distribution (Fig.5C, E and G).

### Mitochondrial localization of iNOS in iNOS-positive A375 cells by Western blot and confocal analyses

**Figure 6:**
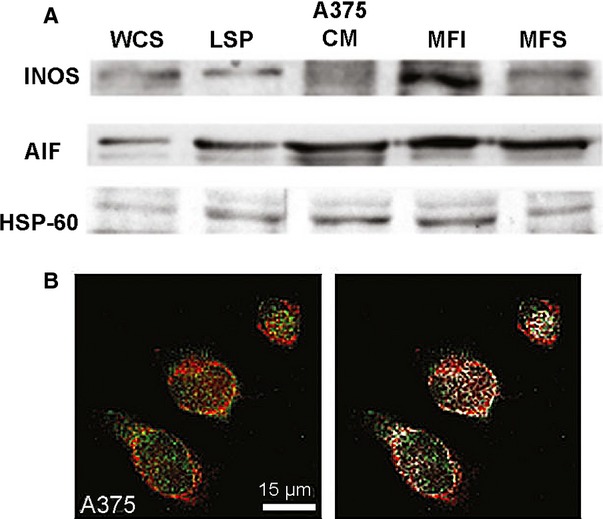
Analysis of expression of iNOS in A375 melanoma cells. (A) iNOS protein expression in different fractions of A375 cells obtained during the preparation of mitochondria by differential centrifugation following purification on an iodoxanol or sucrose gradient as described in the Materials and methods section. WCS: whole cell lysate; LSP: low speed pellets; CM: crude mitochondria; MFI: mitochondrial fraction iodixanol; MFS: mitochondrial fraction sucrose. AIF and HSP-60 were used as mitochondrial marker proteins. One representative experiment out of three performed is shown. (B) Representative confocal immunofluorescence images of A375 melanoma cells grown on glass coverslips, incubated with 100 nmol/l of MitoTracker red CMXRos to label mitochondria (red), fixed and immunostained for iNOS expression (green). Note the colocalization between the two fluorescent signals (yellow) indicating the mitochondrial localization of the iNOS and the qualitative assessment of the colocalized points (white) obtained by using Image J colocalization Plugin Software (NIH). The images are representative of at least three separate experiments with similar results.

To determine whether the presence of iNOS on mitochondrial membrane, as observed in our experiments, only affects P1(0.5) cells, used in these experiments or it is a phenomenon occurring in other iNOS-positive cells, we have determined the expression of iNOS on mitochondrial membrane of melanoma A375 iNOS-positive cells. Western blot analysis revealed the presence of iNOS in mitochondria fractions of A375 cells (Fig.6A). Confocal immunofluorescence confirmed the results obtained by Western blot analysis, showing the presence of iNOS on mitochondrial membrane of these cells (Fig.6B and C).

## Discussion

These data show that human HCC cells that overexpress the MDR1 phenotype constitutively express iNOS in mitochondria as well as P-gp, BCRP and MRP1 in comparison to their parental drug-sensitive cells. The iNOS was expressed in the inner mitochondrial membrane at the level of the cristae; in addition, it was possible to modulate its expression by using drugs, such as the coxib inhibitor celecoxib. However, celecoxib-induced changes of iNOS expression in mitochondria were not because of its inhibitory effect on COX-2 activity but to unknown mechanism. It is possible to say that only low concentrations of celecoxib caused changes in iNOS expression, higher concentration of the coxib, though inhibitory on the COX-2 activity, had no effect of the expression of iNOS. Furthermore, these data show that COX-2 was not expressed in mitochondria although it was present in the cytosol of the same cells. In the same time, it must be said that cells that constitutively express iNOS, as melanoma cells A375, also express iNOS in mitochondria without being MDR1 positive and not expressing P-gp. According to us, this means that occurring of MDR1 phenotype and iNOS expression in mitochondria are not mutually linked.

The expression of iNOS at the inner membrane level of the mitochondrial wall, where proteins are involved in the respiratory chain, suggests a role of iNOS and its product nitric oxide in the transport of electrons at this level. Nitric oxide, produced by the activity of iNOS, is a lipophilic reactive compound with an unpaired electron that can easily diffuse through cell membranes. For this reason, nitric oxide can act inside the cell as a free radical, leading to oxidative stress 21. This increases reactive oxygen species (ROS) production, and cell proliferation and minor O2 utilization by cells 21. As cancer frequently grows in hypoxic conditions *in vivo*, the expression of iNOS at mitochondrial level is advantageous to cancer cells for survival in hypoxic conditions. ROS formation triggers cell proliferation, so all together features of MDR1-overexpressing cancer cells create a vicious circle where increased ROS formation and iNOS expression enhance cancer cell proliferation, leading to hypoxia that causes secretion of VEGF, hepatocyte growth factor and the hypoxia inducible factor with the contemporary enhancement of the MDR1 gene transcription 17,21.

With regard to the modulation of iNOS expression, we observed that low concentrations (2.5 or 5 μmol/l) of celecoxib, that inhibits COX-2 activity, increases iNOS expression, and contemporarily reduces P-gp and BCRP expression in mitochondria. Interestingly, concentration of 10 μmol/l of celecoxib had the opposite effect on iNOS expression, reducing its expression together with that of P-gp, whereas even higher concentration of celecoxib (50 μmol/l) had no effect on P-gp, BCRP or iNOS mitochondrial expression. These observations suggest that it is possible to modulate the expression of iNOS in mitochondria by using drugs. They also suggest that celecoxib can produce different effects on iNOS and P-gp expression, depending upon its concentration. This phenomenon can explain controversial results observed in cancer treatment. Clearly, the effect of celecoxib on iNOS, P-gp and BCRP expression in addition to what we have shown about its capacity to induce apoptosis in MDR1-positive cancer cells could be responsible for its reported anticancer effect 16,33,34.

These data also show that COX-2 is not expressed in the mitochondria of MDR1-overexpressing cancer cells, suggesting that the enzyme is not involved in the regulation of iNOS, P-gp and BCRP expression in mitochondria of these cells. COX-2 is constitutively expressed in the cytosol of these cells, probably as they use prostaglandins as protective compounds in toxic environments. It was also proposed that COX-2 could be involved in the regulation of P-gp expression 12, although this does not seem to be the case in mitochondria 33–35.

Clearly, based on these data, it is possible to say that expression of iNOS and occurrence of MDR1 phenotype in cancer cells are not necessarily linked each to other. Melanoma cell line A375 that express iNOS in the cytosol and mitochondria do not express P-gp and the MDR1 phenotype as shown here. Findings of this work are in agreement with previous results where we showed that celecoxib-induced apoptosis was because of the inhibition of P-gp expression by a COX-2-independent mechanism 16.

In conclusion, the idea that the MDR1 cancer cell phenotype is only because of overexpression of proteins that transport anticancer drugs outside cancer cells has changed somewhat. The expression of proteins such as P-gp, BCRP, MRP1, iNOS and others in any cell membrane, including mitochondria, is much more complex than what was originally thought. This complexity explains the failure of all attempts to overcome cancer cell resistance to any anticancer treatment based on a single drug.
